# Fast Recovery of the Amblyopic Eye Acuity of Kittens following Brief Exposure to Total Darkness Depends on the Fellow Eye

**DOI:** 10.1155/2019/7624837

**Published:** 2019-04-24

**Authors:** Donald E. Mitchell, Elise Aronitz, Philip Bobbie-Ansah, Nathan Crowder, Kevin R. Duffy

**Affiliations:** Department of Psychology & Neuroscience, Dalhousie University, Halifax, NS, Canada B3H 4R2

## Abstract

Recent studies conducted on kittens have revealed that the reduced visual acuity of the deprived eye following a short period of monocular deprivation imposed in early life is reversed quickly following a 10-day period spent in total darkness. This study explored the contribution of the fellow eye to the darkness-induced recovery of the acuity of the deprived eye. Upon emergence of kittens from darkness, the fellow eye was occluded for different lengths of time in order to investigate its effects on either the speed or the extent of the recovery of acuity of the deprived eye. Occlusion of the fellow eye for even a day immediately following the period spent in darkness blocked any recovery of the acuity of the deprived eye. Moreover, occlusion of the fellow eye two days after the period of darkness blocked any further visual recovery beyond that achieved in the short period when both eyes were open. The results imply that the darkness-induced recovery of the acuity of the deprived eye depends upon, and is guided by, neural activity in the mature neural connections previously established by the fellow eye.

## 1. Introduction

The extreme shift of eye preference of neurons in the visual cortex and the accompanying anatomical changes observed in kittens [1], infant monkeys [2], ferrets [3], and rodents [4, 5] that follow a brief postnatal period of monocular deprivation (MD) are widely touted as the quintessential demonstration of developmental cortical plasticity. Accompanying the physiological and anatomical sequelae of MD are severe behavioural deficits that are especially noteworthy in terms of changes to the visual ability of the deprived eye [6]. The consequences of MD occur only during certain critical periods of vulnerability that vary widely in their profile and duration across species. For kittens, susceptibility to the physiological consequences of MD within the visual cortex rises from low levels at the time of natural eye opening at about a week of age to a peak at 4 to 5 weeks followed by a gradual decline to negligible values at an age beyond 4 months but before 8 months [7–9]. Because of the large magnitude and highly reproducible consequences of MD, this form of early deprivation has been employed extensively to study the interaction between programs of gene expression and visually driven neural activity in the development of the central visual pathways (e.g., [10–14]). In addition, MD by eyelid suture has become a standard and convenient means to provide an animal model of human amblyopia and in particular the type referred to as deprivation amblyopia that is typically observed in children with a history of an early opacity of the optical media, such as a cataract, or other peripheral obstruction to clear imagery in one eye.

In addition to documentation of the visual deficits that are observed immediately upon cessation of a period of early MD, there have been many studies of the extent and pace of any recovery that occurs afterward in various situations. The simplest recovery situation, commonly referred to as binocular recovery, is where normal visual input is restored to the deprived eye to allow simultaneous visual input to both eyes without any other manipulation. All other recovery situations include additional manipulations beyond restoration of normal visual input to the deprived eye in an effort to promote greater recovery of the vision of this eye. The simplest of these is reverse occlusion where at the time vision is restored to the deprived eye, the other eye is occluded. The improvement of the visual acuity of the deprived eye with binocular recovery can be substantial but never complete in cats, a result that is in general agreement with the physiological recovery observed in the visual cortex [15, 16]. However, in monkeys, very little behavioural or physiological recovery is observed in this recovery condition [17, 18]. Reverse occlusion can promote fast and substantial recovery of various visual functions of the deprived eye in kittens, but this does not occur without concurrent reduction of the visual abilities of the fellow eye [15, 19]. The reciprocal changes in the vision of the two eyes find a close parallelism with the rapid physiological shifts of ocular dominance following reverse occlusion in the visual cortex of both cats [20, 21] and monkeys [22].

Other recovery conditions, such as the use of mixed daily visual exposure that include adjacent periods of binocular exposure and occlusion of the nondeprived eye [23], have been explored in an effort to prevent some of the unwanted consequences of reverse occlusion [24, 25]. Although mixed daily visual exposure can promote complete recovery of the visual acuity of the deprived eye to normal levels and without any loss of the acuity of the fellow eye, it is effective only under a restricted set of conditions of deprivation and recovery [23].

A promising new recovery condition has been the use of a 10-day period of total darkness that was originally shown to reverse both the behavioural and physiological consequences of an early period of MD in adult Long-Evans rats [26, 27]. More recently, the same period of darkness was found to either prevent the development of amblyopia or promote recovery from amblyopia in kittens induced by a prior period of MD without any ill effects on the acuity of the fellow eye [28, 29]. For these kitten studies, the effect of the period of darkness was examined either when it was imposed immediately after the 7-day period of MD that was initiated at P30 days, or else 8 weeks later when kittens were about 3 months old. In the first situation, the period of darkness resulted in a profound reduction of the vision of both eyes such that the animals appeared blind temporarily following emergence from the darkroom. Thereafter, a slow but matched visual recovery of the two eyes occurred such that the visual acuity of both eyes achieved normal levels in about 50 days. At no time was the acuity of the deprived eye lower than that of the fellow eye so that darkness imposed immediately after the period of MD prevented the development of amblyopia. By contrast, in the second situation when the period of darkness occurred late, the deprived eye was amblyopic at the time darkness was imposed. Remarkably, the acuity of the deprived eye improved very fast after the period of darkness such that normal age-matched levels were achieved in 7-10 days or even less.

The fivefold faster recovery of the acuity of the deprived eye when darkness occurred 2 months after the period of MD as compared to when it followed immediately afterward raises the possibility that visually driven neural activity generated by the fellow eye may make an important contribution to the visual recovery induced by darkness. It was suggested [28–30] that the remarkable behavioural benefits of darkness arose from its ability to promote changes to various key molecules that collectively increase the level of plasticity in the developing visual cortex so as to effectively reset it to a more juvenile and plastic state. The faster rate of recovery of the acuity of the deprived eye following imposition of darkness well after the period of MD, as opposed to immediately afterwards, runs counter to expectations based on the greater age of the animals in the former situation when the level of plasticity would be expected to be much lower. On the other hand, the potential contribution of neural activity generated in cortical neurons by visual stimulation of the fellow eye to the recovery of the vision of the deprived eye after darkness follows explanations linked to the level of the cortical response to visual stimulation of the fellow eye in the immediate aftermath of darkness in the two recovery situations. When darkness is imposed well after the period of MD, neural activity generated by visual stimulation of the fellow eye may serve as a “scaffold” or otherwise guide the reestablishment of neural connections with the deprived eye. However, when the periods of MD and darkness are contiguous, the latter causes a drastic reduction of the vision of the fellow eye [29], and hence, cortical neural activity generated by visual stimulation of this eye would be too low to serve a leadership role in the recovery of the other eye. In the current study, the potential role of neural activity mediated by the fellow eye to the recovery of the deprived eye induced by darkness has been investigated by occluding the fellow eye for various periods of time after termination of darkness.

## 2. Materials and Methods

### 2.1. Animals and Rearing Conditions

The study was conducted on 18 kittens derived from 6 litters that were bred and reared in a closed animal colony at Dalhousie University. The animal colony and all animal procedures followed protocols approved by the Dalhousie University Committee on Laboratory Animals and conformed to the guidelines of the Canadian Council on Animal Care. All 18 animals received a 7-day period of MD at about postnatal day 30 (P30) that was followed in all but 2 animals by a 10-day period of total darkness that began at about 3 months of age (at ~P90). Two animals received a period of occlusion of the fellow eye instead of a period of darkness at an equivalent age. Three control animals chosen from 3 separate litters received a period of darkness without any occlusion of the fellow eye.  Table 1 displays the rearing history of all animals, their gender and, litter of origin.

For all but the period spent in darkness, animals were housed in colony rooms that were illuminated usually on a 12 : 12 h light/dark cycle that was changed to as high as a 14 : 10 cycle in some rooms on occasions to promote breeding. During the day, animals ran free in the colony rooms but at night were housed in large interconnected cages within the colony rooms. The behavioural measurements of acuity that we made did not require any reduction in the amount or nature of their daily food. For the 10-day period of total darkness, animals were moved to a large darkroom (3.8 × 3.5 m) that was part of a darkroom facility that is described in detail elsewhere [31]. The facility contained two adjacent darkrooms that were accessible through several small anterooms and doors that ensured that the two darkrooms were light-tight. To entrain an activity cycle, a radio in the darkroom was automatically turned on and off at times that corresponded to the lighting cycle of the colony rooms. In the darkroom, animals were kept in a large cage (1.5 × 0.7 × 0.9 m) with a 24 cm ledge running the length of the cage. As the animals were 3 months old at the time, they were past the age at which they had been weaned and so were held in the darkroom without their mother. Usually, there was only one or two kittens in the darkroom at the same time and they were held in the same large cage. A second cage was added on the rare occasions in which additional kittens were in the darkroom at the same time.

### 2.2. Surgical Procedures

Monocular deprivation by eyelid suture of the left eye was achieved by use of a two-stage procedure developed [25] to both achieve a secure eyelid closure and allow fast recovery of a normal patent palpebral aperture to facilitate the behavioural assessment of the vision of this eye after the eyelids were opened. As detailed descriptions of these procedures have been provided in a recent paper [32], only a brief summary is provided here. All surgical procedures were performed under gaseous isoflurane anesthesia (2-3% in oxygen), and an s.c. injection of Anafen for postprocedure analgesia was administered once the animals were anesthetized and local anesthesia was administered with Alcaine sterile ophthalmic solution (proparacaine hydrochloride). The first stage of the procedure was to carefully dissect the palpebral conjunctivae free from the upper and lower eyelids and to suture them together with 6-O Vicryl suture thread. A broad-spectrum topical antibiotic (chloromycetin 1%) was then applied to the sutured conjunctivae. The second stage of the surgical procedure was to oppose and suture the exposed tissue on the underside of the eyelids together with 6-O or 5-O silk.

To open the eyelids after the initial week-long period of MD or a later period of occlusion of the fellow eye, animals were anesthetized with isoflurane and any remaining suture material was removed. The eyelids and underlying conjunctivae were then gently cut and pulled apart to achieve a normal palpebral opening. A broad-spectrum topical antibiotic (chloromycetin 1%) was applied to the cornea and surrounding conjunctivae.

### 2.3. Behavioural Measurements of Visual Acuity

Measurements of visual acuity for square-wave gratings were made by use of a jumping stand and procedures developed and refined over the last four decades in this laboratory [16, 25, 31]. The training and testing procedures that are used currently and employed for this study have been described in detail recently [31] and so are summarized only briefly here. The stimuli were adjacent large (19 × 19 cm) horizontal and vertical square-wave gratings of the same period (and, hence, spatial frequency) and with a luminance of 80 cd/m2. Acuity was measured by use of a descending method of limits with jumps to the vertical (positive) stimulus rewarded by food and petting while errors resulted in a denial of these rewards. Because the changes in spatial frequency between blocks of trials were very small and equated on a logarithmic scale with as many as 12 steps/octave change in spatial frequency, only a single trial was provided at the lowest spatial frequencies until an error was made. At this point, the animal had to make 5 consecutively correct responses or a minimum of 7 correct responses out of a maximum 10 trials provided at any spatial frequency, before the spatial frequency was increased. Within about 5 steps of spatial frequency from threshold, the minimum number of trials was increased to 5. Threshold, defined as the highest spatial frequency for which the animal performed at a level of 70% or better, was typically sharp so that performance fell from flawless to chance within 3 step changes of spatial frequency. Kittens exhibited a number of stereotypical behaviours near threshold that included a drastic increase in latency to respond, crying, looks towards nearby objects or to one or both of the people involved in testing, and attempts to back away from the edge of the jumping platform.

Once animals were trained, which usually occurred in the fifth and sixth week, measurements of thresholds were made daily or else every second day. With only a few exceptions, the testing of the acuity was conducted in the morning at about the same time for each individual animal. Two of the authors seated on either side of the jumping platform conducted the tests of acuity with one person providing the food and social reward after each response while the other person recorded the response and prepared the stimuli for the next trial. Tests of the acuity of the deprived eye were made with a hard opaque contact lens occluder placed in the other eye. Six occluders of different base curvatures selected to match the mean corneal curvatures of young kittens of various ages [33] were used as the animals matured. To mitigate against any possible pain, a drop of a local ophthalmic anesthetic (proparacaine hydrochloride 1%) was placed in the eye to be occluded prior to insertion of the contact lens. No signs of any discomfort were evident in the 20 minutes of occlusion of the fellow eye that was typically required for the measurement of the acuity of the deprived eye. Measurement of binocular acuity was used as a substitute for monocular measurement of the grating acuity of the nondeprived eye as in the past, they have been demonstrated to be identical. An additional advantage of this practice was that it enabled measurement to be made of the acuity of the deprived eye immediately afterward as the cornea of this eye would be free from distortion that would accompany the use of the contact lens occluder required for monocular measurement of the nondeprived eye acuity [34]. Following the period of MD, the acuity of the deprived eye gradually improved but eventually reached a stable level that remained so for several weeks prior to the period of darkness. During the time when the acuity of the deprived eye was stable, the starting spatial frequency for each acuity measurement was altered to ensure that the threshold reflected a true visual barrier irrespective of the number of trials or length of the testing session. During the time that the visual acuity of the two eyes had stabilized, the frequency of testing was reduced to once or twice weekly. When possible, more frequent daily tests were reinstated within 10 days of the period of dark exposure.

## 3. Results

### 3.1. Control Animals

**Figure 1:**
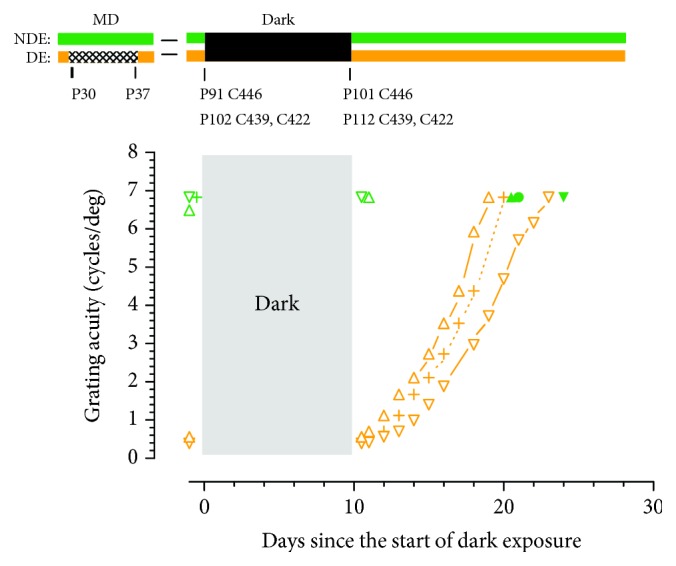
The grating acuity of the two eyes of 3 monocularly deprived kittens immediately prior to and following a 10-day period spent in complete darkness. Each kitten received a 7-day period of MD starting at P30 days and were placed in darkness at either P91 days (C446) or at P102 days (C439, C422). As with subsequent figures, the visual histories and acuity data in the graphs below for the deprived eye (DE) and nondeprived eye (NDE) are depicted in orange and green, respectively. Occlusion of the DE during the early period of MD is shown by crosshatching, and the subsequent period of total darkness is indicated in black. The results of acuity measurements for the 3 animals are depicted by symbols as follows: C422 upright triangles, C446 plus symbols or solid circles (NDE), and C439 inverted triangles. The results of binocular measurements of acuity are shown as open green symbols while monocular measurements of the acuity of the nondeprived eye acuity with which they are equivalent are shown as solid green symbols.

To set the stage for tests of the role of the fellow eye in recovery of the vision of the deprived eye promoted by darkness, two control conditions were necessary. The first of these was a replication of the benefits of darkness conducted on three kittens that were littermates of the animals allocated to the various periods of occlusion of the fellow eye following exposure to darkness. A second control condition was conducted on two animals that had received the same period of MD but had not subsequently been exposed to darkness in order to determine the effects of a period of eyelid occlusion of the fellow eye at an equivalent age to that experienced by the experimental animals. Results from the first of these control conditions in the period immediately prior to and following the 10 days of darkness are displayed together in Figure 1 in order to highlight the similar speed and extent of the darkness-induced recovery of the acuity of the deprived eye observed in all 3 animals. Although two of the animals (C422 and C439) were placed in darkness at almost the same age (at P101 or P102), the third (C446) was so exposed earlier at P91. In agreement with the results obtained in two prior studies [28, 29], the acuity of the deprived eye of all three animals recovered fast to match that of the other eye within either 9 (C422), 10 (C446), or 13 (C439) days. There did not appear to be any simple relationship between the speed of recovery of the deprived eye and the age at which darkness was imposed as one of the animals exposed at P101 recovered almost as fast as the one exposed earliest at P91 (C446). Because all three animals were derived from different litters, it is possible that the slight differences in the rate of recovery could be attributed to differences between litters.

**Figure 2:**
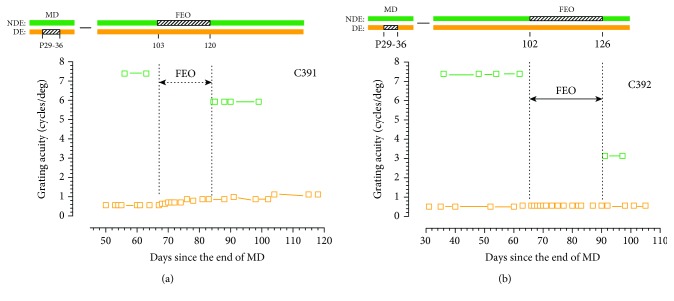
The grating acuity of the deprived eye (orange symbols) and the binocular acuity (green symbols) of 2 monocularly deprived littermate kittens prior to and following 17 (a) (C391) or 24 (b) (C392) days of fellow eye occlusion (FEO). The visual histories of the two animals are depicted above the two graphs.

Although it has been known from many earlier studies that occlusion of the fellow eye can result in an improvement of the visual acuity of the deprived eye of kittens following a prior period of MD, in all but a few isolated animals, the manipulation occurred immediately adjacent to the initial period of MD [15, 19, 24]. A similar rearing protocol had been adopted in prior electrophysiological investigations of the ability of fellow eye occlusion to reverse the effects of a preceding period of MD on cortical ocular dominance [20, 21]. Possibly because occlusion of the fellow eye followed immediately after termination of the period of MD in these studies, it was referred to as reverse occlusion. In order to establish the effects of a period of occlusion of the fellow eye made several months *after* the initial period of MD, two littermate control animals were reared with such occlusion imposed at an equivalent age to that of the experimental animals at the end of their period of exposure to darkness. Both animals received an initial 7-day period of MD at P29 that was followed by either a 17- (C391) or 24- (C392) day period of occlusion of the fellow eye at P103 and P102 days, respectively. From prior studies, it was anticipated that occlusion of the fellow eye at this late age would not produce substantial improvement of the acuity of the deprived eye or negatively impact the acuity of the fellow eye. The results, displayed in Figure 2, provided only partial support for these expectations. In one animal (C391), occlusion of the fellow eye resulted in a very small improvement of the acuity of the deprived eye, from 0.56 to 0.87 cycles/deg, while for the other animal (C392), the acuity of this eye remained unchanged despite a longer period of fellow eye occlusion. On the other hand, the acuity of the fellow eye was reduced in both animals from 7.4 to 5.93 cycles/deg for C391 and to 3.14 cycles/deg for C392 representing losses of 0.32 and 1.23 octaves, respectively. The asymmetric changes in the acuity of the two eyes following fellow eye occlusion that were particularly noteworthy for C392 may reflect physiological changes in the visual cortex reported initially by Mioche and Singer [35] following MD or reverse occlusion. In both situations, the initial change was a decrease of the excitatory response to the newly deprived eye followed by a much slower increase in the response to the other eye. It is not unreasonable to suppose that similar electrophysiological events would be observed in the slightly different situation employed here where occlusion of the fellow eye was delayed with respect to termination of the initial period of MD.

### 3.2. Experimental Animals

**Figure 3:**
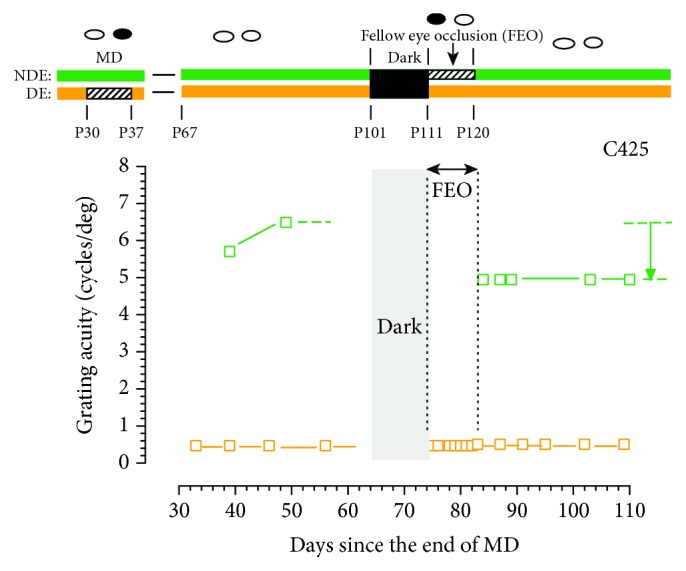
The grating acuity of the deprived eye (orange symbols) and the binocular acuity (green symbols) of a monocularly deprived kitten (C425) surrounding a 10-day period of total darkness followed by a 9-day period of fellow eye occlusion (FEO). As with the previous figures, the visual histories of the two animals are depicted in schematic form by the bars and icons above the two graphs.

**Figure 4:**
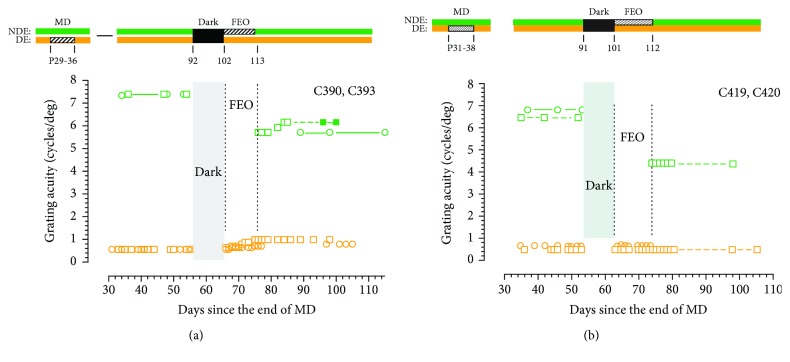
The grating acuity of the deprived (orange symbols) and nondeprived (green solid symbols) eyes as well the binocular acuity (green open symbols) of two pairs of monocularly deprived kittens surrounding 10 days of total darkness followed immediately by an 11-day period of fellow eye occlusion (FEO). As with previous figures, the visual histories of the animals are depicted in schematic form by the bars above the two graphs. (a) Data for C390 (square symbols) and C393 (circle symbols). (b) Data for littermates C419 (square symbols) and C420 (circle symbols). The period of FEO effectively blocked any improvement of the acuity of the deprived eye following the period of darkness but did cause a reduction of the acuity of the nondeprived eye.

**Figure 5:**
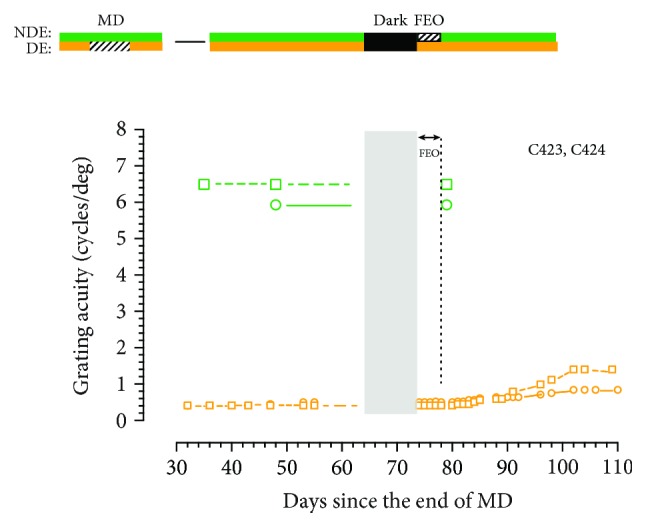
The grating acuity of the deprived eye acuity (orange symbols) and the binocular acuity (green symbols) of two monocularly deprived littermates C423 (square symbols) and C424 (circle symbols) surrounding 10 days of total darkness followed immediately by a 4-day period of fellow eye occlusion (FEO). Whereas the period of FEO effectively blocked any improvement of the acuity of the deprived eye following the period of darkness, it did not result in any reduction of the acuity of the nondeprived eye.

The initial experiments were conducted on five animals with 9-11-day periods of occlusion of the fellow eye immediately following the interval spent in darkness. This occlusion time was chosen as it corresponded to that required for the acuity of the deprived eye to recover to normal levels in two previous studies when both eyes were open following darkness [28, 29]. The results for the animal (C425) that received only 9 days of occlusion of the fellow eye are shown in isolation in Figure 3, while those for the 4 animals that received 11 days occlusion of this eye are displayed as pairs in Figures 4 and 5 in order of the dates when they were tested. For all 5 animals, data are shown beginning about a month prior to the period of darkness. The data for C425 are displayed with elaborate schematic descriptors of the animal's rearing history to aid comprehension of the more sparse representations used in subsequent graphs. For C425, the rearing history is represented both by the icons at the very top and by the horizontal bars that illustrate the four major periods of visual exposure (binocular visual exposure, MD, darkness, and occlusion of the fellow eye) as well as the postnatal ages at which they began and ended. The data for C425 revealed that following darkness, there was no change in the acuity of the deprived eye during the 9-day period of occlusion of the fellow eye. Moreover, following the period of occlusion of the fellow eye, the acuity of this eye was reduced from 6.5 to 4.95 cycles/deg or 0.4 of an octave. There was no change in the visual acuity of either eye in the ensuing month. This result stands in marked contrast to the data of Figure 1 where following the period of darkness, the acuity of the deprived eye recovered from about 0.5 cycles/deg to 6.8 cycles/deg or an improvement of 3.8 octaves without any negative impact on the acuity of the fellow eye.

A very similar pattern of results was observed in the first two kittens that received a slightly longer 11-day period of occlusion of the fellow eye immediately following dark exposure. For C390 (Figure 4(a)), the combination of darkness and fellow eye occlusion resulted in only a very slight overall improvement of the acuity of the deprived eye from 0.56 to 1.0 cycles/deg or 0.84 octaves that was accompanied by a small reduction of the acuity of the fellow eye. During the period of occlusion of the fellow eye following darkness, the acuity of the deprived eye improved very little, from 0.55 to 0.7 cycles/deg or 0.35 octaves. It was not possible to measure the acuity of the deprived eye of the other animal (C393; Figure 4(a)) for almost 3 weeks following the period of occlusion of the fellow eye due to persistent conjunctival inflammation of this eye. Following treatment with ophthalmic topical antibiotics, all signs of inflammation disappeared so that short-term occlusion of this eye with an opaque contact lens could be resumed to allow measurements of the acuity of the deprived eye once more. However, the acuity of this eye did not change appreciably in the ensuing weeks. The period of occlusion of the fellow eye following darkness resulted in a similar decline of the acuity of the nondeprived eye to that observed for C390. Fellow eye occlusion of the two animals reared most recently; C419 and C420 (Figure 4(b)) also blocked any recovery of the acuity of the deprived eye following darkness. One animal (C420) was found dead early on the morning of the penultimate day of intended occlusion of the fellow eye, and the necropsy performed by the university veterinarian revealed that the presumptive cause of death was a congenital ventricular septal defect. Although the unexpected premature death of C420 prevented assessment of the effect of the period of fellow eye occlusion on the acuity of this eye, the consequences of fellow eye occlusion for its littermate C419 were substantial as the acuity of this eye was reduced from 6.5 to 4.38 cycles/deg or 0.57 octaves.

Occlusion of the fellow eye for 9-11 days blocked any recovery of the acuity of the deprived eye following the period of darkness in all 5 animals, not just during the period of occlusion but in the days that followed. The absence of any change in the acuity of the deprived eye following fellow eye occlusion implies that any heightened plasticity induced in the central visual pathway by exposure to darkness does not extend beyond 11 days as the acuity of the deprived eye remained unchanged following restoration of binocular visual exposure. This result prompted us to explore the consequence of a much shorter 4-day period of fellow eye occlusion imposed on two littermates. The results displayed in Figure 5 indicate that the short period of fellow eye occlusion blocked any recovery of the acuity of the deprived eye after darkness during the period of occlusion and in its immediate aftermath. However, about 4 days later and for the next month, the acuity of the deprived eye began a slow improvement that was most evident for C423 as the acuity tripled from 0.4 to 1.4 cycles/deg or a change of 1.8 octaves. However, the improvement observed in its littermate, C424, was much smaller. This result suggests that some residual darkness-induced plasticity may have remained after termination of fellow eye occlusion to allow some limited recovery of the acuity of the deprived eye. No decrement in the acuity of the fellow eye was observed after the 4 days of occlusion. Again, the most prominent feature of the results from the two animals was the extent to which occlusion of the fellow eye blocked the benefits for the acuity of the deprived eye of the preceding period of darkness. The result implies that binocular visual input in the immediate aftermath of the period of dark exposure may be essential to the recovery of the acuity of the deprived eye. This possibility was explored on two additional groups of kittens for which the fellow eye was occluded for either 1 day or 2 days.

**Figure 6:**
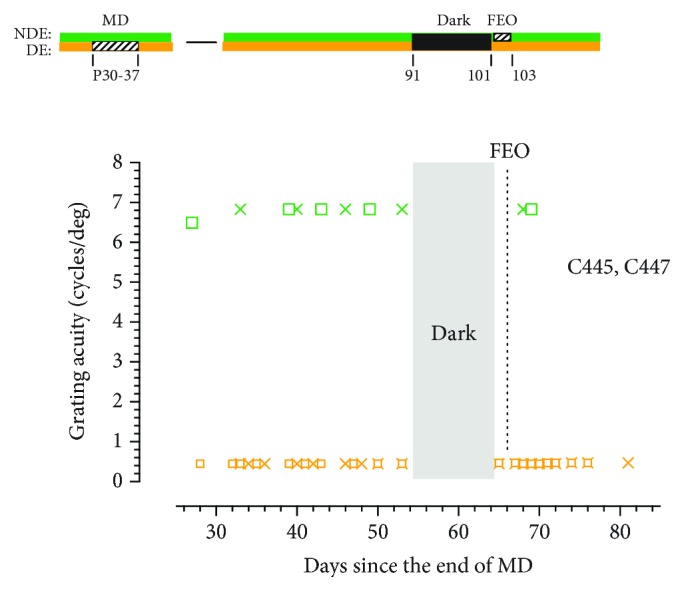
The grating acuity of the deprived eye acuity (orange symbols) and the binocular acuity (green symbols) of two monocularly deprived littermates C445 (square symbols) and C447 (cross symbols) surrounding 10 days of total darkness followed immediately by a 2-day period of fellow eye occlusion (FEO). Whereas the period of FEO effectively blocked any improvement of the acuity of the deprived eye following the period of darkness, it did not result in any reduction of the acuity of the nondeprived eye.

**Figure 7:**
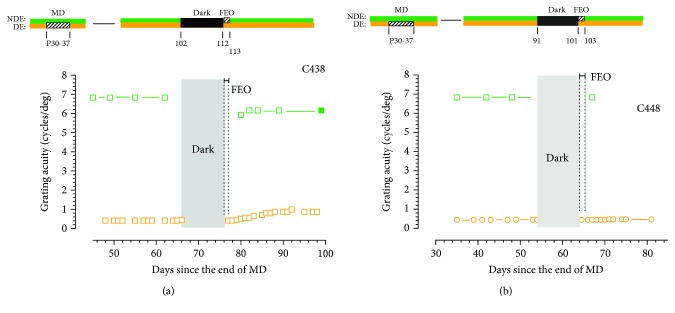
The grating acuity of the deprived eye acuity (orange symbols) and the binocular or nondeprived eye acuity (green symbols) of two monocularly deprived kittens C438 (a) and C448 (b) surrounding 10 days of total darkness followed immediately by a 1-day period of fellow eye occlusion (FEO). The brief period of FEO effectively blocked any improvement of the acuity of the deprived eye following the period of darkness.

The results from the two animals (C445 and C447) for which the fellow eye was occluded for 2 days (Figure 6) indicate that this short period of occlusion blocked any recovery of the visual acuity of the deprived eye at all in the next 3 weeks. Also, the short period of occlusion of this eye did not cause any reduction in the acuity of this eye once occlusion was terminated. Two further animals were reared with just one day of occlusion of the fellow eye that was initiated immediately after the period of darkness. Because the two animals were exposed to darkness at different ages, their data has been plotted separately in Figure 7. Even one day of occlusion of the fellow eye had an adverse effect on the recovery of the visual acuity of the deprived eye. In one animal, C438, the acuity of the deprived eye recovered a little from 0.4 to 0.9 cycles/deg but the acuity of this eye showed no improvement at all for the other animal (C448). Together, the results from the 11 animals that had the fellow eye occluded immediately upon emergence from the darkroom implied that concordant binocular visual exposure may be essential in the first 24 hours after darkness for the latter to promote recovery of the acuity of the deprived eye.

**Figure 8:**
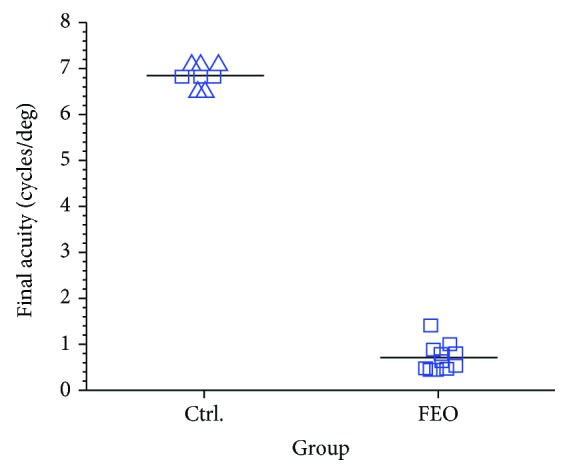
The open square symbols show a comparison of the final acuity achieved by the deprived eye of the 3 animals in the control group (Ctrl) following darkness with that achieved by the deprived eye in the 11 animals that had the fellow eye occluded for various short periods of time after they were removed from the darkroom. The triangle symbols for the control group display the results from animals reared in an identical fashion in two prior studies ([28]; C151, C152, C155, and C157) and [29]; C304).

The increase in deprived eye acuity was poorly correlated with the duration of fellow eye occlusion following darkness (*r*^2^ = 0.03; *p* = 0.64). Therefore, the adverse consequence of fellow eye occlusion was summarized in Figure 8 by comparing the final acuity achieved by the amblyopic eye among the 11 animals with fellow eye occlusion following darkness with that of control animals from this and two earlier studies [28, 29] that had both eyes open after darkness. The mean (±s.d.) deprived eye final acuity of the animals in the control group (6.85 ± 0.25 cycles/deg) was substantially higher than that achieved by animals in the fellow eye occlusion group (0.71 ± 0.30 cycles/deg). A permutation test for a difference in the mean final acuity between these two groups was highly significant (*p* < 0.00001).

**Figure 9:**
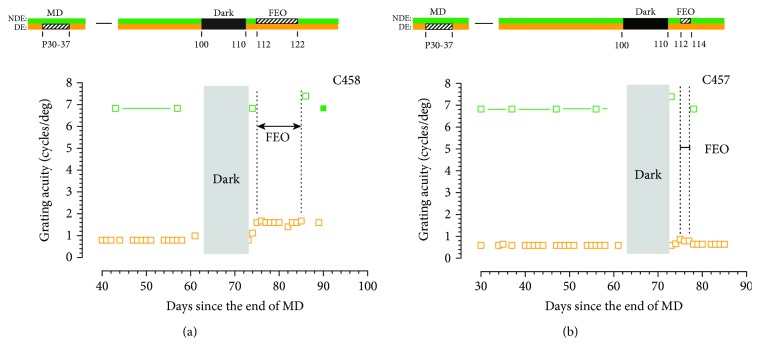
The grating acuity of the deprived eye (orange symbols) and the binocular or nondeprived eye acuity (green symbols) of two monocularly deprived kittens C458 (a) and C457 (b) surrounding 10 days of total darkness followed immediately by a 2-day period of binocular visual exposure that preceded fellow eye occlusion (FEO) for either 10 or 2 days. The period of FEO effectively blocked any further improvement of the acuity of the deprived eye that began during the brief period of binocular visual exposure following the period of darkness.

In a preliminary exploration of the importance for binocular visual input in the immediate aftermath of the 10-day exposure to darkness, two additional animals were reared with a 2-day period of binocular visual exposure interposed immediately prior to either a 2- (C457) or 10- (C458) day period of occlusion of the fellow eye. The different periods of occlusion of the fellow eye employed for these animals dictated that their data be plotted separately in Figure 9. Despite the different occlusion times for the fellow eye, the pattern of results was very similar. For both animals, the acuity of the deprived eye improved during the short 2-day period of binocular exposure that followed exposure to darkness by an amount similar to that observed in the same period by the 3 control animals of Figure 1 that had both eyes open after 10 days exposure to total darkness. However, occlusion of the fellow eye prevented any further improvement in the acuity of the deprived eye.

## 4. Discussion

As replicated again on three additional animals in this current study (Figure 1), a 10-day period of darkness can promote fast recovery of the visual acuity of the deprived eye of monocularly deprived kittens to match that of the fellow eye. It remains to be seen whether darkness restores other consequences of MD for the vision of the deprived eye such as on contrast sensitivity functions or on vernier acuity. However, there is evidence that darkness can restore normal stereoscopic vision to about a third of kittens following exposure to darkness [29].

Leaving aside for now examination of the ability of darkness to promote complete recovery of all visual functions of the deprived eye, the goal of this study was to investigate the contribution of the fellow eye to the darkness-induced recovery of the visual acuity of the amblyopic eye of monocularly deprived kittens. To test for a crucial role of the fellow eye in this recovery process, the rather blunt intervention of monocular eyelid suture of this eye was used to severely degrade the spatial visual information contained in the retinal image of this eye and so constrain the ability of neural signals from this eye to reinforce signals from the amblyopic eye at synapses in the visual cortex. The results from the first group of animals that received 9-11 days of occlusion of the fellow eye immediately following 10 days exposure to total darkness were very clear as they demonstrated that such occlusion blocked completely any recovery of the acuity of the amblyopic eye. Because the length of the occlusion extended through the time to total recovery of the acuity of the amblyopic eye that is observed when both eyes are open after the period of darkness (Figure 1), it was thought possible that some recovery may occur after shorter periods of fellow eye occlusion due to possible partial persistence of the benefits of the prior dark period. Progressively shorter periods of fellow eye occlusion of 4 days (Figure 5), 2 days (Figure 6), and even one day (Figure 7) effectively blocked any substantial recovery of the acuity of the deprived eye (Figure 8). An obvious conclusion that could be drawn is that binocularly concordant visual input in the immediate aftermath of the period of darkness is essential for the latter to be effective. Stated differently, if neural activity from the fellow eye is discordant with that from the amblyopic eye on the first day after the animal is removed from total darkness, it cannot bootstrap the rapid improvement of the acuity of the amblyopic eye.

The result from the final set of animals displayed in Figure 9 suggests a rather more nuanced conclusion. Here, the fellow eye was occluded two days *after* animals were removed from the darkroom for either 2 (C457) or 10 (C458) days. Nevertheless, occlusion of this eye stopped any further recovery from that achieved over the two days of binocular visual input experienced immediately after the period of darkness. The fact that recovery was blocked by just 2 days of occlusion of the fellow eye (C457) implies that brief occlusion *at any time after darkness* and not just in its immediate aftermath can block recovery and further that normal binocular vision is required throughout the recovery process. Occlusion of the fellow eye appears to be an extremely potent way to disrupt the benefits of darkness for recovery from early monocular deprivation.

Although occlusion of the fellow eye represents a rather blatant way to disrupt the degree of concordance of the visual input to the two eyes, it will be important to investigate whether less severe forms of disrupted binocular visual input such as those introduced by prismatic deviation of the two visual axes or by refractive imbalance can block the benefits of darkness in the same decisive manner as that achieved by fellow eye occlusion. It will also be of interest to determine if recovery following dark exposure is also prevented by complete elimination of retinal activity from the fellow eye, which can be achieved through intraocular administration of tetrodotoxin [32, 36]. The degradation of normal vision experience through the closed eyelid of the fellow eye may be worse for the recovery of the amblyopic eye than a complete elimination of vision in the fellow eye.

The speed of recovery of the deprived eye that follows a period of darkness suggests that it cannot be explained entirely by the formation of new axonal or dendritic connections with this eye in the visual cortex. Whatever the underlying mechanisms for darkness-induced recovery, it appears to be prompted and guided in some way by neural activity generated by the fellow eye. Upon emergence from the darkroom, the strong cortical neural activity generated by the fellow eye may initially reinforce residual and/or silenced neural connections with the deprived eye in a behaviourally meaningful way that could precipitate further modifications to promote recovery and consolidate gains in visual acuity. The dramatic consequences of occlusion of the fellow eye described here provided strong support for this idea. That this recovery could be blocked by very short periods of occlusion, even after it had begun (Figure 9), deserves further exploration.

### 4.1. Clinical Implications

The results of fellow eye occlusion may hold important clinical applications with respect to the use of an interval of dark exposure, or possibly binocular retinal inactivation [36], to treat amblyopia in humans. For either treatment to be successful, it is essential that binocular visual input be concordant and remains so in the immediate aftermath of the treatment. In particular, optimal recovery precludes the use of patching of the fellow eye following the period of darkness or possibly binocular retinal inactivation. From a clinical perspective, it is clearly desirable to establish whether darkness or binocular retinal inactivation can prompt recovery from early monocular deprivation in primates. Because of ethical and practical barriers to the use of darkness, it is likely that initial attempts will employ brief binocular retinal inactivation. An additional practical barrier to a study on primates, particularly with respect to the consequences of either treatment for vision, is the present lack of methods to provide fast behavioural assessments of visual thresholds. However, it is possible to employ alternative measures such as the use of cortical visually evoked potentials (VEPs) to assess the effects on vision. In addition, the benefits of darkness or binocular retinal inactivation can be assessed in terms of their anatomical effects on the lateral geniculate nucleus [32].

## Figures and Tables

**Table 1:** Animals and rearing conditions. Timing (postnatal days of age) of the initial period of monocular deprivation (MD), darkness, and subsequent visual exposure (fellow eye occlusion and/or binocular vision) for all 18 kittens. Details are also provided on the 6 litters and the gender of the kittens. Prior to and for the 8+ weeks following the period of MD, all kittens received binocular visual exposure.

Animal	Litter ID	Gender	MD	Darkness	Fellow eye occlusion (no. of days)	Binoc. vision
Control animals						
*Darkness only*						
C422	D	F	P30-37	P101-111		
C439	E	F	P30-37	P102-112		
C446	F	M	P30-37	P91-101		
*Fellow eye occlusion only*						
C391	B	M	P29-36		P103-120 (17 d)	
C392	B	M	P29-36		P102-126 (24 d)	

Experimental animals						
*Darkness followed by fellow eye occlusion*						
C425	C	M	P30-37	P101-111	P111-120 (9 d)	P120-
C390	A	F	P29-36	P92-102	P102-113 (11 d)	P113-
C393	B	F	P29-36	P92-102	P102-113 (11 d)	P113-
C419	D	F	P31-38	P90-100	P100-111 (11 d)	P111-
C420	D	F	P31-38	P91-101	P100-111 (11 d)	
C423	C	M	P30-37	P101-111	P111-115 (4 d)	P115-
C424	C	F	P30-37	P101-111	P111-115 (4 d)	P115-
C445	F	F	P30-37	P91-101	P101-103 (2 d)	P103-
C447	F	F	P30-37	P91-101	P101-103 (2 d)	P103-
C448	F	F	P30-37	P91-101	P101-102 (1 d)	P102-
C438	E	M	P30-37	P102-112	P112-113 (1 d)	P113-
*Darkness followed by a short period of binoc. vision then fellow eye occlusion*						
C457	G	M	P30-37	P100-110	P112-114 (2 d)	P110-112; P114-
C458	G	F	P30-37	P100-110	P112-122 (10 d)	P110-112; P122-

## Data Availability

The data used to support the findings of this study are available from the corresponding author upon request.
